# Millimeter-wave radar object classification using knowledge-assisted neural network

**DOI:** 10.3389/fnins.2022.1075538

**Published:** 2022-12-22

**Authors:** Yanhua Wang, Chang Han, Liang Zhang, Jianhu Liu, Qingru An, Fei Yang

**Affiliations:** ^1^Radar Research Laboratory, School of Information and Electronics, Beijing Institute of Technology, Beijing, China; ^2^Beijing Institute of Technology Chongqing Innovation Center, Chongqing, China; ^3^Advanced Technology Research Institute, Beijing Institute of Technology, Jinan, Shandong, China; ^4^Electromagnetic Sensing Research Center of CEMEE State Key Laboratory, School of Information and Electronics, Beijing Institute of Technology, Beijing, China; ^5^Beijing Rxbit Electronic Technology Co., Ltd., Beijing, China

**Keywords:** millimeter-wave radar, object classification, knowledge-assisted, neural network, artificial intelligence

## Abstract

To improve the cognition and understanding capabilities of artificial intelligence (AI) technology, it is a tendency to explore the human brain learning processing and integrate brain mechanisms or knowledge into neural networks for inspiration and assistance. This paper concentrates on the application of AI technology in advanced driving assistance system. In this field, millimeter-wave radar is essential for elaborate environment perception due to its robustness to adverse conditions. However, it is still challenging for radar object classification in the complex traffic environment. In this paper, a knowledge-assisted neural network (KANN) is proposed for radar object classification. Inspired by the human brain cognition mechanism and algorithms based on human expertise, two kinds of prior knowledge are injected into the neural network to guide its training and improve its classification accuracy. Specifically, image knowledge provides spatial information about samples. It is integrated into an attention mechanism in the early stage of the network to help reassign attention precisely. In the late stage, object knowledge is combined with the deep features extracted from the network. It contains discriminant semantic information about samples. An attention-based injection method is proposed to adaptively allocate weights to the knowledge and deep features, generating more comprehensive and discriminative features. Experimental results on measured data demonstrate that KANN is superior to current methods and the performance is improved with knowledge assistance.

## Introduction

Thanks to the complex structure and mechanisms of the brain, humans have the capability to continuously learn new knowledge, perceive complex environments, and make precise decisions (Cornelio et al., 2022; Kuroda et al., 2022). With the groundbreaking discovery of cells and continuous research in neuroscience, a variety of artificial neural networks have been proposed (van Dyck et al., 2022). Neural networks have promoted the development of artificial intelligence (AI) technologies in many fields, such as smart healthcare (Alsubai et al., 2022; Soenksen et al., 2022), intelligent transportation (Zhu et al., 2019; Zhu F. et al., 2020), etc. Similar to humans, networks acquire capabilities through learning. However, they learn things by brute force optimization based on input data, which limits their performance in various practical applications. To promote the next generation of AI technology, the neurology mechanism of the human brain learning process is studied, and the brain mechanism or knowledge is integrated into neural networks to help networks improve the perception and understanding of the world (Marblestone et al., 2016; Lindsay, 2020; Zhu J. et al., 2020).

This paper mainly focuses on the application of AI technology in advanced driving assistance system (ADAS), which has proved its effectiveness in safe driving and its evolution is in full swing. To elaborately capture the surroundings, multiple sensors are equipped on vehicles, such as cameras, LiDAR and millimeter-wave (MMW) radar. Cameras provide high-resolution optical images that are in line with human visual cognition and are widely applied in object detection (Redmon et al., 2016; Ren et al., 2017; Kim and Ro, 2019; Deng et al., 2022) and tracking (Danelljan et al., 2014; Smeulders et al., 2014; Nam and Han, 2016; Zhao et al., 2017; Han et al., 2019b) tasks. Although cameras offer optical images and give a semantic understanding of real-world scenarios, it is not robust facing adverse conditions, such as weak lighting or bad weather (Wang et al., 2021). As for LiDAR, it generates point cloud data and can be utilized for object detection and localization (Qi et al., 2018; Shi et al., 2019, 2021). However, these methods require dense point clouds to describe detailed information for accurate prediction, and LiDAR also has poor robustness to fog (Bijelic et al., 2018), rain or snow.

Compared with cameras and LiDAR, MMW radar is more reliable and robust in harsh environments. It is widely used in many practical scenarios, such as remote sensing target detection and classification (Liu et al., 2018; Wang et al., 2018; Liu et al., 2021; Tang et al., 2022) and intelligent transportation (Munoz-Ferreras et al., 2008; Felguera-Martin et al., 2012). Therefore, perception from pure radar data becomes a valuable alternative (Wang et al., 2021). Although it is widely used to obtain accurate location information about different objects (Prophet et al., 2019), it is still a challenge to extract discriminative semantic features from radar data for precise object classification. Great efforts have been made to advance MMW radar object classification performance. Existing researches are mainly based on three kinds of radar data, including micro-Doppler signatures (Villeval et al., 2014; Angelov et al., 2018; Held et al., 2019), point clouds (Feng et al., 2019; Zhao et al., 2020) and range-Doppler (RD) maps or range-azimuth maps (Major et al., 2019; Palffy et al., 2020). Since RD maps can be easily obtained in engineering and maintain rich Doppler and object motion information, this paper focuses on object classification based on RD maps.

Typical feature-based approaches (Rohling et al., 2010; Heuel and Rohling, 2012) extract hand-crafted features from RD maps, such as velocity, extension in range dimension, etc., which are physically interpretable. Then, a support vector machine (SVM) classifier is trained to classify the features. To extract these features, humans constantly learn and summarize laws from various objective things and construct feature extraction algorithms based on accumulated knowledge and experience. Therefore, these methods heavily rely on human experience and algorithm design, and their performance may degrade in complex practical application scenarios.

Recent advances in deep learning have promoted the development of automatic object classification. By learning and optimizing details from pure input data, neural networks can accomplish various specific tasks. A convolutional neural network (CNN) has been established to extract valuable features for automotive radar object classification (Patel et al., 2019; Shirakata et al., 2019). Recently, a radar object detection method was proposed (Gao et al., 2019), which combines a statistical constant false alarm rate (CFAR) detection algorithm with a visual geometry group network 16 (VGG-16) classifier (Simonyan and Zisserman, 2015). After that, RadarResNet (Zhang A. et al., 2021) was constructed for dynamic object detection based on range-azimuth-Doppler maps. Ouaknine et al. (2021) utilized a fully convolutional network (FCN) to accomplish object detection and classification tasks. A RODNet (Wang et al., 2021) was proposed for radar object detection based on cross-modal supervision approach. These methods automatically learn features from training data and obtain good results. However, they discard human knowledge, which means the information they obtain may be not comprehensive.

In order to promote the intelligence of radar object classification and achieve more accurate and stable performance, it is a trend to introduce prior knowledge generated from human brains and experience into neural networks for assistance and guidance. Recently, similar ideas and methods have been studied in many fields. Reference (Chen and Zhang, 2022) presented the concept of knowledge embedding in machine learning and summarized the current research results. In the field of radar target classification, physics-aware features were obtained from synthetic aperture radar (SAR) images and injected into the layer of a deep network to provide abundant prior information for training and classification (Huang et al., 2022). In Zhang et al. (2022), azimuth angle and phase information were extracted from SAR images and served as domain knowledge to improve the performance of SAR vehicle classification. For polarimetric high-resolution range profile classification, a feature-guided network was proposed with state-of-the-art results (Zhang L. et al., 2021). In the driving assistance system, the information obtained by the tracker has been studied to improve object classification accuracy (Heuel and Rohling, 2011). A state-aware method was proposed to model the discrimination and reliability information synchronously into the tracking framework to ensure robust performance (Han et al., 2019a).

Following the idea, a knowledge-assisted neural network (KANN) using RD map sequence for automotive MMW radar object classification is proposed. The primary intention is to inject knowledge into the neural network to supplement the network with physical information and to improve the classification performance. The network imitates the structure of neural mechanisms in human brains, however, it achieves learning tasks through brute force optimization of input data and lacks perception of the practical physical world. While knowledge is generated based on how and what the human brain thinks when accomplishing complex tasks. It conforms to human brain cognition and is an objective and physical description of the objects in the practical world. By fusing the knowledge and high dimensional data fitting, the network will have some physical cognition capability and be more similar to the way the human brain perceives, which will improve the network performance in practical driving assistance applications.

Specifically, in the method, the RD map sequence is served as input, which consists of several frames of region-of-interest (RoI) about an object based on CFAR algorithm. To improve the performance of the network, two kinds of prior knowledge of RD maps based on human expertise are extracted and hierarchically integrated into the network for assistance. The first one is image knowledge which describes the explicit spatial information of the RD map. It is obtained from the algorithms consistent with the human brain visual mechanism and applied to the attention mechanism to help the network more accurately concentrate on object regions. The second one is object knowledge which represents the semantic attribute information of objects. It includes the ranges, velocities, azimuths, and RD map extension features, which are important when humans are classifying objects. Additionally, RD maps of the same object may vary with different ranges, velocities, etc. Therefore, object knowledge is injected into the network to assist its training and classification. It is combined with the deep features extracted from the network adaptively through an attention-based injection method to provide more comprehensive and discriminative features. Experimental results on measured data of four kinds of objects demonstrate that KANN can achieve advanced performance and the assistance of knowledge is helpful.

## Knowledge-assisted neural network

**FIGURE 1:**
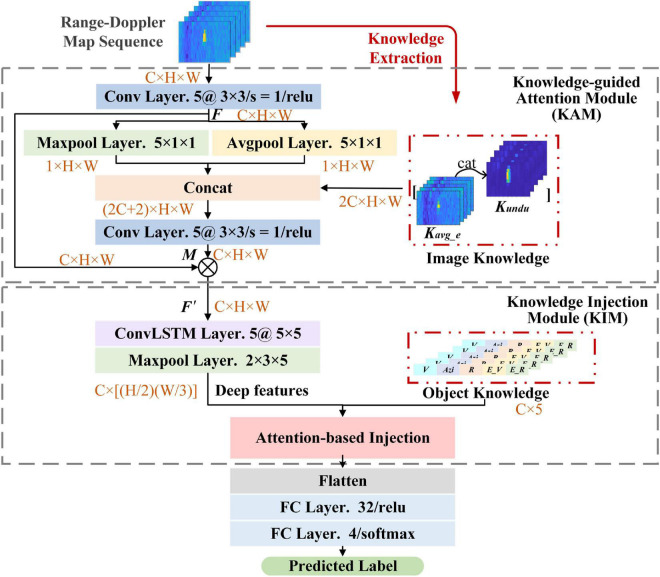
Illustration of knowledge-assisted neural network (KANN). (C, H, and W denote the channel number, height, and width of the data; ⊗ represents Hadamard product).

The architecture of KANN is shown in Figure 1. KANN employs the RD map sequence containing several consecutive frames of RoIs in RD maps about an object as input data. The RoIs are cut out from RD maps based on CFAR algorithm. Different frames are fed into the network as different channels to provide temporal dimension information. Knowledge-guided attention module (KAM) and knowledge injection module (KIM) are proposed to generate the features for classification with knowledge assistance. The knowledge utilized is some prior information obtained from artificial algorithms, and it contains the physical cognition consistent with human brain when humans classify objects in traffic environments. Specifically, in KAM, an attention mechanism is established, and the prior image knowledge containing specific spatial information is applied to help make the attention assignment more reasonable and discriminative. KIM is utilized to extract spatiotemporal information about input data. Inspired by the human brain cognition when classifying objects, in this module, object knowledge containing semantic attribute information is adaptively injected into the network to provide more valuable information for classification. The rest of this section will first introduce the RD map sequence generation method in detail. Then, the specific contents of KAM and KIM are explained.

### Range-Doppler map sequence generation

**FIGURE 2:**
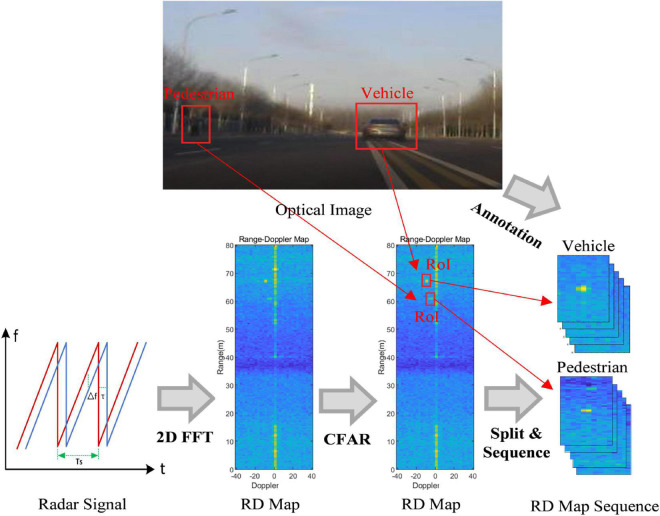
The workflow of range-Doppler (RD) map sequence generation.

MMW radar dominantly transmits continuous chirps and receives the reflected echoes from objects. The workflow of RD map sequence generation is shown in Figure 2. First, the RD map is generated from the radar signal through the 2D-fast Fourier transform (FFT). The 2D-CFAR detection algorithm is then utilized to detect the objects. After that, inspired by Patel et al. (2019), a fixed-size RoI of the RD map is cut out for each detected object. Finally, an RD map sequence is constructed by stacking several RoIs about the same object in continuous frames of RD maps. The RoIs across frames are associated based on the range and velocity correlation, which means the detection results with the largest overlap are regarded as the same object. It should be noted that, to provide temporal information, in a sequence, the highest detected peak of the object is in the center of the first RoI, and the rest RoIs have the same location in the RD map as the first one. The ground truth categories of the RD map sequences are annotated according to the optical images. Specifically, before the data collection, the radar sensor and camera are calibrated in typical scenarios. First, the range and azimuth measurement results of the radar sensor are calibrated based on angle reflectors. Then, some cooperative pedestrians, cyclists, and cars are employed as detection objects on a test road. The radar data and optical images are recorded separately, and the locations and other information of the objects from the two sensors are compared and calibrated. Finally, after collecting the measured data, the RoIs in RD maps are labeled based on the optical images.

### Knowledge-guided attention module

Since an object only occupies a small region in the RD map, KAM establishes an attention mechanism that is inspired by the visual attention mechanism of human brains (Lindsay, 2020). It generates different weights to help networks focus on the discriminative regions in each RoI, while suppressing unnecessary ones. In KAM, as shown in Figure 1, image knowledge is prepared as the assistant knowledge to participate in the generation of the attention matrix for more precise attention assignment. Considering that the spatial information obtained by the network lacks the physical cognition of the practical world, introducing image knowledge can make the network assign attention in a way more similar to the human brain. The image knowledge is obtained from algorithms based on human expertise and is composed of the average energy (*K^avg_e^*) and undulation feature (*K^undu^*) which delineate the exact spatial information and distinguish the target and background clutter. Given a pixel *s*_*ij*_ whose location is (*i*, *j*) in an RoI, we consider the pixels in the surrounding region with the size of 3 × 3 to calculate the features:


(1) $$K_{i\,j}^{a\,v\,g\,\_\,e}=\frac{1}{n}\,\left(\sum_{i-1}^{i+1}\sum_{j-1}^{j+1}s_{i\,j}^{2}\right),$$

(2) $$K_{i\,j}^{u\,n\,d\,u}=\frac{1}{n}\,\left(\sum_{i-1}^{i+1}\sum_{j-1}^{j+1}{\left(s_{i\,j}-\bar{s}\right)}^{2}\right),$$

where *n* = 9 and $\bar{s}$ denotes the number of pixels and the mean amplitude of the RoI, respectively. By stacking the two feature sequences in channel dimension, image knowledge of the RD map sequence can be obtained.

**FIGURE 3:**
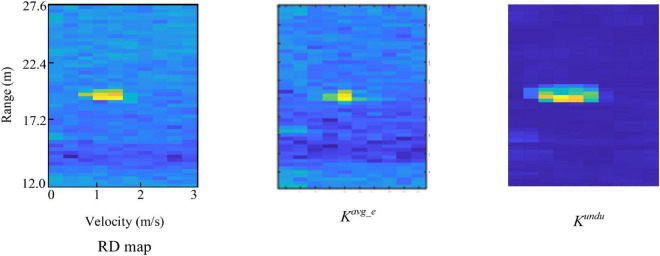
*K^avg_e^* and *K^undu^* extracted from the same region-of-interest (RoI) in an range-Doppler (RD) map.

Figure 3 shows the two features extracted over the same RoI in an RD map. It can be observed that *K^avg_e^* and *K^undu^* can represent the spatial information consistent with the visual cognition of the human brain. Concretely, *K^avg_e^* describes the average energy of the region and highlights the target regions, while *K^undu^* describes the amplitude undulation information.

In this module, given an RD map sequence, it is first processed with a convolutional layer whose kernel size is 3 × 3 to obtain the feature map ***F***. Then, a max pooling layer and an average pooling layer are applied to down-sampled ***F*** in two aspects to capture spatial information autonomously. The size of the layers is configured to 5 × 1 × 1 to obtain compact spatial information. At this time, image knowledge is introduced to concatenated with the pooling results in channel dimension to generate the weight matrix ***M***:


(3) M=σ⁢(f⁢(c⁢o⁢n⁢c⁢a⁢t⁢(M⁢a⁢x⁢P⁢o⁢o⁢l⁢(F);A⁢v⁢g⁢P⁢o⁢o⁢l⁢(F);I⁢M⁢K))),

where σ denotes the “relu” activation function, *f* represents the convolution operation, *MaxPool* (⋅) and *AvgPool* (⋅) denote the max pooling and average pooling operation respectively. Next, according to ***M***, the redefined feature map ***F***′ can be obtained:


(4) $$F^{\prime }=M⊗F,$$

where ⊗ denotes Hadamard product.

Compared with most existing attention mechanisms, KAM improves the physical perception ability of the network and can explore more accurate attention distribution by embedding image knowledge which is obtained from human expertise and contains precise spatial information of samples.

### Knowledge injection module

Since the RoI from a whole RD map only represents a portion of the radar field-of-view, the network trained with the data will lack the radial velocity, range, and other information of objects in the real world. However, for the same object, the shape or extension in the RD map may vary with its velocity, range, and azimuth relative to the radar sensor. Missing this information can lead to poor classification performance of the network. Therefore, to generate more discriminative features for classification, in KIM, object knowledge is injected into the network by combining with the deep features. Object knowledge includes the velocity, range, azimuth, range profile (*P*_*r*_), and velocity profile (*P*_*v*_) of the object in each RoI. These five kinds of information not only offer real-world information about the objects, but also have the capability of classification (Prophet et al., 2018). In this way, the network can improve the overall perception of samples, which is more similar to the cognition of the human brain and can enhance the performance of the semantic classification task.

**FIGURE 4:**
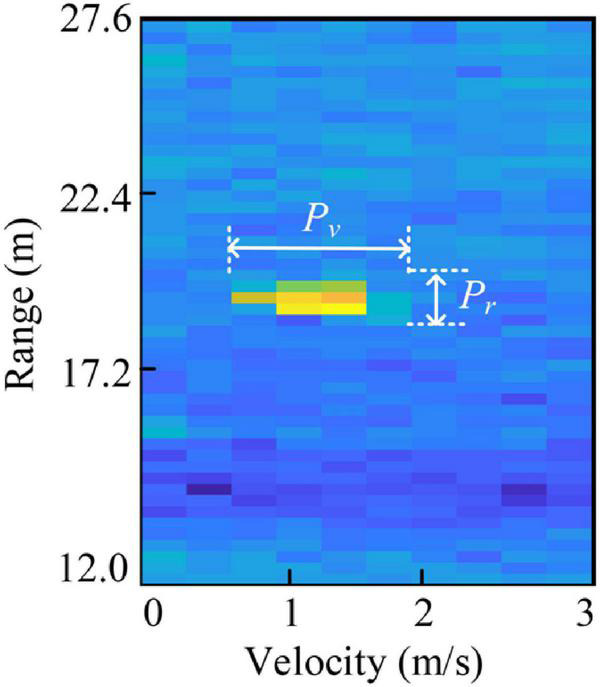
The schematic diagram of *P*_*r*_ and *P*_*v*_.

The velocity, range, and azimuth can be obtained by 3D-FFT. From the RD map obtained by 2D-FFT, the radial range and relative velocity can be captured. Then, FFT is performed on the range-velocity bins to estimate the azimuth. *P*_*r*_ and *P*_*v*_ describe the target extensions in range and velocity dimensions, as shown in Figure 4. *P*_*r*_ and *P*_*v*_ are the maximum length of detected points in range and velocity dimensions of the object, respectively, and can be calculated by:


(5) $$P_{r}=\left(r_{e}-r_{s}+1\right)\cdot \Delta \,R,$$

(6) $$P_{v}=\left(v_{e}-v_{s}+1\right)\cdot \Delta \,v,$$

where *r*_*s*_ and *r*_*e*_ denote the starting and ending points detected, Δ*R* represents the range resolution. In (6), *v*_*s*_, *v_*e*_*, and Δ*v* denote the similar meanings in velocity dimension.

The structure of KIM is given in Figure 1, a ConvLSTM (Shi et al., 2015) layer is employed to extract the deep features containing spatiotemporal information. ConvLSTM network is a recurrent structure owing good capability of modeling sequential data and extracting temporal information. Meanwhile, it can learn the spatial information of each individual time step due to the convolution operation. Therefore, considering that the input is a sequence, ConvLSTM network is suitable for extracting deep features from both temporal and spatial dimensions simultaneously.

**FIGURE 5:**
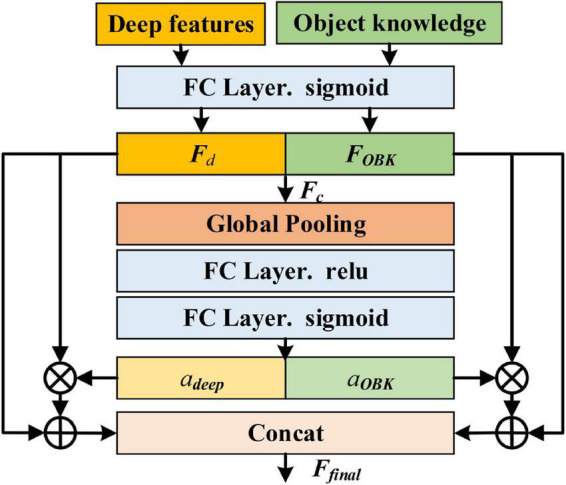
Illustration of attention-based injection method (⊗ represents Hadamard product; ⊕ denotes element-wise addition).

Then, object knowledge is combined with deep features. Considering that there is a gap between the two features and the same feature may have different contributions in different classification tasks, inspired by squeeze-and-excitation networks (Hu et al., 2018), we adopt an attention-based method to combine object knowledge and deep features in an adaptive way, as shown in Figure 5. Specifically, the deep features and object knowledge are first mapped to the same dimension through an fully connected (FC) layer and scaled to 0∼1 by sigmoid activation, respectively, making them similar and conducive for combination. Then, the mapped features, ***F***_*d*_ and ***F***_*OBK*_, are connected in channel dimension and ***F***_*c*_ can be acquired. After that, the global pooling operation is utilized to squeeze ***F***_*c*_, and two FC layers are adopted to learn the attention weight vector ***a*** containing two elements:


(7) $$\text{a}=\left[a_{O\,B\,K},a_{d\,e\,e\,p}\right]=\delta \,\left({\text{W}}_{2}\cdot \sigma \,\left({\text{W}}_{1}\cdot A\,v\,g\,P\,o\,o\,l\,\left({\text{F}}_{c}\right)\right)\right),$$

where *a*_*deep*_ and *a*_*OBK*_ are the weights of the deep features and object knowledge, respectively, δ and σ are “sigmoid” and “relu” activation functions, ***W***_2_ and ***W***_1_ are parameter matrices, *AvgPool* (⋅) denotes the average pooling operation. Subsequently, object knowledge and the deep features are redefined by multiplying with the corresponding weights. Next, ***F***_*d*_ and ***F***_*OBK*_ are added to their redefined results to preserve original information from different sources. Finally, the concatenated features are used for classification:


(8) $${\text{F}}_{f\,i\,n\,a\,l}=c\,o\,n\,c\,a\,t\,\left({\text{F}}_{d}+a_{d\,e\,e\,p}\,{\text{F}}_{d},{\text{F}}_{O\,B\,K}+a_{O\,B\,K}\,{\text{F}}_{O\,B\,K}\right),$$

In this module, by injecting object knowledge, the network trains with sufficient information about samples, which improves its learning capability and classification performance.

## Experiment

In this section, to evaluate the performance of KANN, we conduct a variety of experiments based on a measured dataset. The dataset is first introduced in detail. Then, the classification performance of KANN is assessed by comparative experiments. Additionally, we analyze the influence of the knowledge assistance and network structure on the performance of KANN through experiments.

### Dataset preparation and implementation details

The measured dataset is collected by an automotive MMW multiple-input multiple-output (MIMO) radar with 4 Tx and 8 Rx producing a total of 32 virtual antennas. It uses the Frequency Modulated Continuous Waveform (FMCW) which is widely used in automotive radar (Hu et al., 2019). The specific configurations of radar are provided in Table 1.

**TABLE 1:** The specific configurations of radar.

Parameter	Value
Center frequency and bandwidth	77 GHz and 600 MHz
Maximum range, resolution	80 m, 0.3 m
Maximum radial velocity, resolution	40 m/s, 0.3 m/s
Field of view	−45°∼45°
Number of chirps per frame	64
Number of samples per chirp	256

**FIGURE 6:**
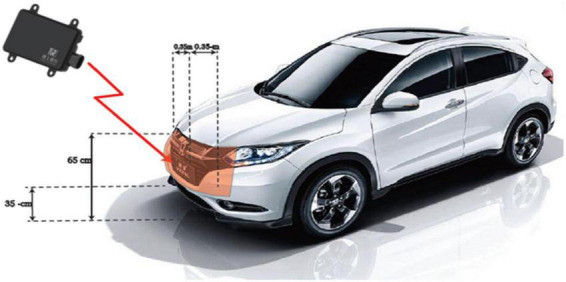
Radar installation diagram.

**FIGURE 7:**
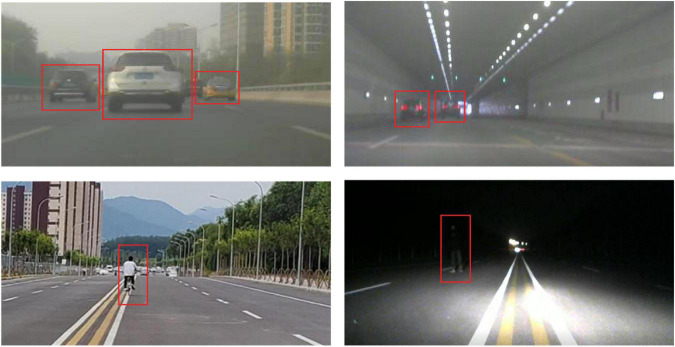
Samples of different data collection scenarios.

The radar sensor is assembled and mounted on the front of the car as shown in Figure 6. The data is collected under different lighting conditions in different scenarios, such as city streets, elevated roads, and tunnels. Some sample scenarios are shown in Figure 7. Four kinds of objects are considered, including pedestrian, runner, vehicle, and cyclist, with overlapping speed ranges.

**FIGURE 8:**
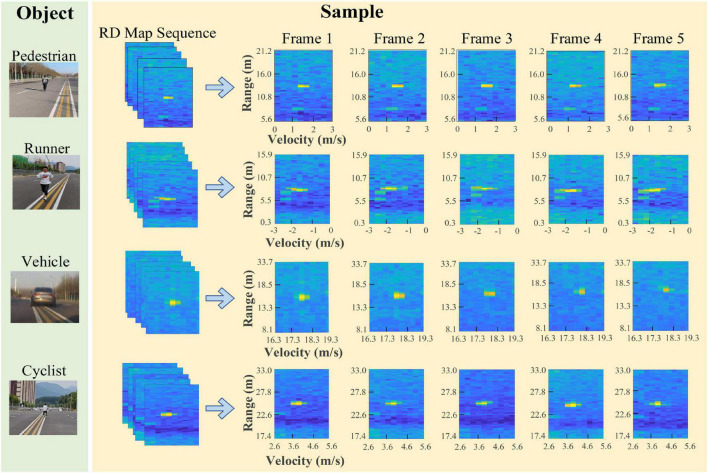
Samples of four kinds of objects.

After collecting the original radar echoes, we perform the sequence generation method and knowledge extraction algorithms to obtain the RD map sequences and two kinds of knowledge. It should be noted that in the experiments we stack the RoIs of the same object in the RD maps of five continuous frames to construct an RD map sequence, and the RoIs in different RD map sequences are completely different. There are some samples given in Figure 8. Then, the samples are randomly divided into training and testing datasets. The detailed settings are listed in Table 2.

**TABLE 2:** The detailed settings of the dataset.

Object	Number of training data	Number of testing data
Pedestrian	840	279
Runner	873	350
Vehicle	1,085	368
Cyclist	714	246
Total	3,512	1,243

Moreover, the implementation details are shown. The experiments are conducted on a server cluster with a 64-bit Linux operating system. In the training phase, the batch size is set to 64, the learning rate is 0.01, and the network is optimized with adaptive moment estimation (Adam) algorithm.

### Evaluation metrics

In order to evaluate the performance of different methods, the average accuracy (AA) of all classes is applied. This metric takes into account the imbalance of the data and can provide a more objective assessment of performance. It can be calculated by:


(9) $$\text{AA}=\frac{1}{C}\,\sum_{c=1}^{C}\frac{N_{T\,P}}{N_{c}},$$

where *C* is the number of classes, *N*_*TP*_ is the number of samples classified correctly in class *c*, and *N*_*c*_ is the total number of samples in class *c*.

### Quantitative analysis

#### Object classification

In this part, we assess the performance of KANN based on the measured dataset. Additionally, six methods that have been studied in this field are served as comparisons. Two of them are feature-based methods that extract the features contained in object knowledge and then utilize SVM (Heuel and Rohling, 2012) and random forest (RF) classifiers to predict the object labels, respectively. The remaining four comparison methods establish different neural networks to accomplish the task, containing Three Layer-CNN (TL-CNN) (Patel et al., 2019), VGG-16 (Gao et al., 2019), FCN (Ouaknine et al., 2021), and RadarResNet (Zhang A. et al., 2021).

The per-class accuracy and AA of different methods are shown in Table 3. The values in bold in the table are the highest accuracy among the methods. We can observe that KANN can achieve advanced performance, and its AA and per-class accuracy is all above 90%. These results demonstrate that KANN is effective in the MMW radar object classification task. Since the two kinds of knowledge are obtained from the human brain’s wisdom and logic, integrating them can inspire the network to learn the samples in a way more similar to the human brain and extract more discriminative and comprehensive features.

**TABLE 3:** Experimental results on different methods.

Method	Accuracy (%)				
	Pedestrian	Runner	Vehicle	Cyclist	AA
Features+SVM **(Villeval et al., 2014)**	60.93	76.13	91.70	91.60	80.09
Features+RF	78.49	67.90	91.70	88.55	81.66
TL-CNN **(Patel et al., 2019)**	83.52	71.19	91.27	90.08	84.02
VGG-16 **(Gao et al., 2019)**	88.53	79.42	93.01	93.13	88.52
FCN **(Ouaknine et al., 2021)**	87.81	**92.59**	94.32	94.66	92.35
RadarResNet **(Zhang A. et al., 2021)**	**94.98**	85.60	**96.51**	90.84	91.98
KANN	93.19	91.35	**96.51**	**95.42**	**93.39**

In order to assess the performance of KANN in practical application, the runtime of different methods is analyzed, and the results are listed in Table 4. It can be observed that the feature-based methods cost the shortest time because their input is a set of prepared artificial feature vectors but not raw data. Among the deep learning methods, the runtime of KANN is the longest, which is 2.053 s. It can be inferred that the structure of ConvLSTM contained in KANN costs more time compared with convolution operation due to its serial units. As for the application, in general, it is an acceptable efficiency for the proposed method and its accuracy is the highest.

**TABLE 4:** Computational costs on different methods.

Model	Runtime (s)
Features+SVM **(Villeval et al., 2014)**	0.013
Features+RF	0.007
TL-CNN **(Patel et al., 2019)**	0.081
VGG-16 **(Gao et al., 2019)**	0.864
FCN **(Ouaknine et al., 2021)**	0.079
RadarResNet **(Zhang A. et al., 2021)**	0.293
KANN	2.053

#### Ablation study

To investigate the advantage of knowledge assistance, we conduct the ablation experiment. The basic network of KANN without knowledge assistance is regarded as the baseline. Then, image knowledge and object knowledge, are separately added to the baseline for assistance. KANN is compared with these three models. Moreover, to assess the structure of KANN, we exchange the positions of KAM and KIM to conduct the experiments. Besides, considering that the spatial information can also be obtained by convolutional operation, in KANN, we remove the image knowledge and apply two convolution kernels with randomly initialized parameters to extract spatial information to compare the effect of artificial image knowledge and the automatically obtained spatial information. The results are listed in Table 5. The values in bold in the table are the highest accuracy among the methods.

**TABLE 5:** Experimental results of the ablation study.

Model	Accuracy (%)				
	Pedestrian	Runner	Vehicle	Cyclist	AA
Baseline	78.92	68.91	87.77	83.97	79.89
Baseline + image knowledge	79.93	73.25	90.83	90.08	83.52
Baseline + object knowledge	81.83	86.83	90.39	92.76	87.95
Baseline + image knowledge + object knowledge (KANN)	93.19	**91.35**	**96.51**	**95.42**	**93.39**
KIM + KAM	83.87	81.84	90.83	92.37	90.53
KANN with convolution operation	**98.57**	84.77	87.77	82.44	88.38

It is shown that the baseline performs worst. When one kind of knowledge is added, the accuracy improves. The integration of image knowledge increases AA by 3.63%, and object knowledge makes the network achieve an 8.06% increase in AA. KANN with image knowledge and object knowledge achieves the best performance, whose AA is more than 10% higher than the baseline. Additionally, we can observe that when KIM lies in the front, AA drops by about 3%. As for the comparison of artificial image knowledge and spatial information from convolution operation, it can be seen that the AA of the model with artificial image knowledge is approximately 5% higher than the model with learnable spatial information. It can be inferred that though the network can automatically extract the information, the artificial features can supplement the network with physical and discriminative information which the network lacks.

From the results, we can conclude that with the knowledge assistance, the network is inspired to learn sample information no longer solely by optimizing data. It can explore information in a way more like the human brain. Although an attention mechanism is built to help the network focus on the object regions first, it is trained by the network learning mechanism. Image knowledge contains sample spatial information, which is acquired based on human brain wisdom and logic. By introducing image knowledge into the attention mechanism, the network can assign attention not only based on the network learning results, but also according to the visual cognition of the human brain. As a result, the network concentrates more precisely on the object region and achieves more accurate classification performance. As for object knowledge, it provides semantic information about objects in the real world, which is in line with the human brain perception when classifying objects. Adding this information supplements more information for the network and can improve the accuracy. Besides, by contrast, object knowledge plays a more significant role in KANN. It can be inferred that object knowledge offers the semantic information which the network lacks, while image knowledge modifies the attention weight matrix. On the other hand, from the module location experiment, it can be inferred that the network can achieve better performance by focusing on the object regions in the early stage and delicately combining object knowledge and deep features through the attention-based method just before classification.

#### Comparison of combination methods

In this part, to evaluate the performance of the attention-based combination method in KIM, different combination methods are applied to the fusion of object knowledge and deep features, and the results are discussed. Two common approaches, including concatenation and element-wise addition are served as the comparison methods. The results are listed in Table 6, we can observe that three combination methods can all achieve good results with AA all above 90%. The values in bold in the table are the highest accuracy among the methods. The experiment proves that the object knowledge definitely supplements physical and discriminative information for the network and improves the classification performance. Among them, the attention-based method performs best because the network can adaptively assign weights of different features, and the features generated are more suitable for this classification task. As for the other two, they are just the simple combination of the two features, and their accuracy is lower than our adaptive attention-based combination method.

**TABLE 6:** Experimental results of different combination methods.

Combination method	Accuracy (%)				
	
	Pedestrian	Runner	Vehicle	Cyclist	AA
Concatenation	90.32	90.53	92.14	93.13	91.53
Element-wise addition	89.96	86.83	94.76	90.84	90.59
Attention-based method	**93.19**	**91.35**	**96.51**	**95.42**	**93.39**

## Conclusion

In this paper, we propose a knowledge-assisted network KANN based on RD map sequence for automotive MMW radar object classification. We introduce two kinds of prior knowledge to help the network learn information from samples in a way more similar to the human brain. In this way, the neural network can generate more discriminative features for semantic classification tasks. Specifically, image knowledge helps the network more accurately focus on the object regions. Object knowledge is fused with the deep feature from the network to provide more comprehensive information for classification. To effectively combine the two aspects of information, an attention-based injection method is employed to achieve the adaptive combination. Experiments based on measured data of four classes of objects verify the effectiveness of KANN and demonstrate that knowledge assistance can improve the performance of the network. Our research is continuing, and the data in more complex traffic scenarios, e.g., the crowded situation and strong interference conditions, is still being collected and processed. Since some researches show that introducing knowledge into the network can mitigate the network data size dependence (Zhang L. et al., 2021; Huang et al., 2022), in future research, based on our expanded dataset, we will conduct further experiments to assess the effect of knowledge injection with the training data size as the main topic. Simultaneously, the practical application value will be further evaluated with the data in more complex traffic scenarios.

## Data availability statement

The datasets presented in this article are not readily available because the dataset is part of ongoing work. Requests to access the datasets should be directed to LZ, zhangliang@bit.edu.cn.

## Author contributions

YW first proposed the idea that introducing prior knowledge into neural networks for assistance, participated in the construction of KANN, analyzed the effectiveness of KANN, and wrote the original manuscript. CH established the network, carried out the experiments based on the measured dataset, and participated in the writing of the original manuscript. LZ organized a study in the field of automotive MMW radar object classification based on knowledge assistance, proposed the detailed framework of KANN, investigated the feasibility of the method, and reviewed the manuscript and made valuable suggestions. JL constructed the measured dataset, arranged the data collection experiments, and reviewed the manuscript. QA and FY conducted the automotive MMW radar data collection experiments. All authors contributed to the article and approved the submitted version.
